# Expression Pattern of Nitric Oxide Synthase during Development of the Marine Gastropod Mollusc, *Crepidula fornicata*

**DOI:** 10.3390/genes12020314

**Published:** 2021-02-22

**Authors:** Marta Truchado-Garcia, Filomena Caccavale, Cristina Grande, Salvatore D’Aniello

**Affiliations:** 1 Departamento de Biología, Facultad de Ciencias, Universidad Autónoma de Madrid, C/Darwin, 1, Cantoblanco, 28049 Madrid, Spain; mtruchado@berkeley.edu; 2 Department of Molecular and Cell Biology, University of California, Berkeley, CA 94720, USA; 3 Biology and Evolution of Marine Organisms, Stazione Zoologica Anton Dohrn, Villa Comunale, 80121 Napoli, Italy; filomena.caccavale@szn.it

**Keywords:** NOS, nitric oxide, evo-devo, evolution, metamorphosis, mollusc

## Abstract

Nitric Oxide (NO) plays a key role in the induction of larval metamorphosis in several invertebrate phyla. The inhibition of the NO synthase in *Crepidula fornicata*, a molluscan model for evolutionary, developmental, and ecological research, has been demonstrated to block the initiation of metamorphosis highlighting that endogenous NO is crucial in the control of this developmental and morphological process. *Nitric Oxide Synthase* contributes to the development of shell gland, digestive gland and kidney, being expressed in cells that presumably correspond to FMRF-amide, serotoninergic and catecolaminergic neurons. Here we identified a single *Nos* gene in embryonic and larval transcriptomes of *C. fornicata* and studied its localization during development, through whole-mount in situ hybridization, in order to compare its expression pattern with that of other marine invertebrate animal models.

## 1. Introduction

The metamorphosis of competent larvae of marine organisms is under endogenous inhibitory control. Several experimental pieces of evidence demonstrated that Nitric Oxide (NO), an endogenous gaseous molecule, is key for the induction of larval metamorphosis in several invertebrate phyla. NO is considered a potent inhibitor of this developmental process acting at nanomolar concentration, implying that its cellular homeostasis needs to be finely regulated, as shown in polychaete annelid *Capitella teleta* [1], in sea urchin *Lytechinus pictus* [2] and in sea squirt *Ciona intestinalis* [3], *Boltenia villosa* and *Cnemidocarpa finmarkiensis* [4]. Noteworthy, the only exceptions found so far to this general statement, given the limited number of species studied, occurs in the poriferan *Amphimedon queenslandica* [5] and in the solitary tropical ascidian, *Herdmania momus*, in which it has been shown that NO has instead a positive regulatory role [6].

Moreover, the role of NO has been extensively studied in the phylum Mollusca and, similar to other phyla, it clearly exerts a predominant function as a negative regulator of metamorphosis, as reported in the hard-shelled mussel *Mytilus coruscus* [7], the slipper shell snail *Crepidula fornicata* [8], the eastern mudsnail *Ilyanassa obsoleta* [9], the sea slug *Alderia willowi* [10], and the Pacific oyster *Crassostera gigas* [11] (Table 1). Nevertheless, in other molluscs NO has been implicated in many other biological functions, such as development and neurotransmission [12,13], immune response [14,15,16], feeding behavior and chemosensory activation [13,17], olfaction [18,19,20] and stress response [21] (Table 1).

All this testifies to the wide and complex tasks that endogenous NO plays in many biological processes. NO is mainly generated by the well-known oxidoreductase enzyme called *Nitric Oxide Synthase* (*Nos*), in which protein structure and functional domains are highly conserved in animal evolution [22]. Moreover, many animals (i.e., molluscs, echinoderms, cephalochordates and vertebrates) have independently duplicated the *Nos* gene [22,23]. Nevertheless, it is still poorly studied if only one or both Nos paralogs in an organism actively contribute to the process of metamorphosis through the production of NO.

In the present work, we identified a single ortholog of *Nos* in embryonic and larval transcriptomes of the marine gastropod *C. fornicata* (*Cfo-Nos*), an emergent laboratory animal model, and we described its expression pattern during development. In a previous elegant work, Pechenik and collaborators [8] demonstrated that endogenous NO controls larval metamorphosis in this species with the use of pharmacological treatments designed to inhibit Nos. Moreover, they highlighted Nos protein localization in apical ganglion, a neural structure implicated in the control of metamorphosis [24]. Therefore, the aim of the present work was to deepen our understanding of *Nos* gene expression patterns during the entire development of *C. fornicata* through whole-mount in situ hybridization (WMISH), unlike previous works that used universal antibodies for Nos protein localization.

## 2. Materials and Methods 

### 2.1. Animal Care and Embryos Fixation 

Adults of the dextral snail *C. fornicata* were harvested from local waters near Woods Hole (USA) by the Marine Resources Center at the Marine Biological Laboratory (MBL). Embryos were collected and reared as previously described [25]. The egg masses have a cluster of grapes-conformation, going from a few to a dozen clear bags. These bags contain around 50 individual and naked embryos. To grow the embryos until the proper stage, the bags forming the egg mass were individualized and incubated at 20 °C in filtered sea water (FSW) with antibiotics (Penicillin/Streptomycin). Embryos and larvae were fixed as described by Perry et al. [26]. The bags were opened under the scope using forceps. The embryos were washed in FSW and fixed in 4% formaldehyde in FSW for one hour at room temperature. After fixation, embryos were washed 3 times in 1x PBS, and dehydrated by 2 washes in 100% MeOH. Embryos were stored at −20 °C until use. 

### 2.2. Sequence Mining and Analysis 

In order to identify candidate *Nos* genes we performed TBlastn searches using the Nos proteins of *Aplysia californica* and *Lottia gigantea* as query sequences. A unique *Nos* gene was retrieved from *C. fornicata* RNA-seq databases generated in Grande’s lab [26,27] from early cleavage stage embryos up to veliger larval stages prior to hatching. RNA-seq databases have been uploaded to Geneious v6.1.2 [28]. 

### 2.3. Cloning and Sequencing 

mRNA was isolated from different developmental stages (cleavage to larvae) of the snail *C. fornicata* and stored in RNAlater (Life Technologies, Carlsbad, CA,USA) at −80 °C. High quality total RNA was extracted using TriZol (Life Technologies, Carlsbad, CA,USA) and purification methods followed previously described protocols [27]. The purity and concentration of total RNA was verified with a NanoDrop ND-1000 Spectrophotometer (Thermo Scientific, Waltham, MA, USA), and cDNA was generated with SMARTer PCR cDNA Synthesis kit (Clontech, Mountain View, CA, USA), following the manufacturer’s protocol. *C. fornicata Nos*-specific primers were designed: Forward: Cfo-NosF CTCACGACACAGAATGTGTTGAGG, and Reverse: Cfo-NosR: AGGAGGGATTTCAAACAGTTCGG, Internal Forward: Cfo-NosFin AATGTGTTGAGGCCGATCCTGACC, and Internal Reverse: Cfo-NosRin: CAAACAGTTCGGGATCCTGACCAG. They were used to PCR amplify a 761 bp fragment using a Platinum Taq DNA polymerase (Invitrogen, Carlsbad, CA, USA). Amplified PCR products were run on 1.5% agarose gel, purified with Zymoclean Gel DNA Recovery kits (ZymoResearch) and cloned into pGEM-T Easy vector (Promega, Madison, WI, USA). Several clones were checked by Sanger sequencing (Secugen SL), and sequences were assembled using FinchTV v.1.4.0 (Geospiza, Seattle, WA, USA), MacVector v12.7 [29] and Sequencher v.5.0 (Gene Codes Corporation, Ann Arbor, MI, USA).

### 2.4. Gene Expression Analysis

DIG-labelled riboprobes for *Cfo-Nos* with 761 bp length were generated. Linearized DNA template (amplified from plasmid DNA with T7/SP6 primers) was used to synthesize sense and antisense DIG-labelled riboprobes with T7 and SP6 RNA polymerases (Life Technologies, Carlsbad, CA, USA), respectively. Riboprobes were purified with RNA Clean & Concentrator-5 kit (ZymoResearch, Irvine, CA, USA), and their concentrations were measured on a NanoDrop ND-1000 Spectrophotometer. WMISH was performed as previously described by Perry and colleagues [26], and specimens were stored at 4 °C in 80% glycerol/20% 1× PBS, until imaged.

### 2.5. Microscopy

Slides were prepared as previously described [26], coating the slides and the coverslips with Rainex. After drying out at room temperature, Kimwipes are used to wipe the residue. The embryos were mounted *in toto* with clay on the corners. For bright field imaging, embryos were visualized on a Zeiss Axioskop 2 Plus microscope (Carl Zeiss, Oberkochen, Germany), and a Coolsnap FX colour camera (Roper Scientific, Trenton, NJ, USA) was used with the Metavue 7.10 program (Molecular Devices, San Jose, CA, USA). Multifocal stacks of bright field images were combined and flattened using Helicon Focus stacking software 6.7.1 (Helicon Soft, Kharkiv, Ukraine). ImageJ (v2.0.0-rc-56/1.51h) and Adobe Photoshop CS5, extended v12.1 x64, were used for processing the embryo images.

## 3. Results and Discussion

### 3.1. One Ortholog for Nos with Neural Signatures Is Present in C. fornicata

**Figure 1:**
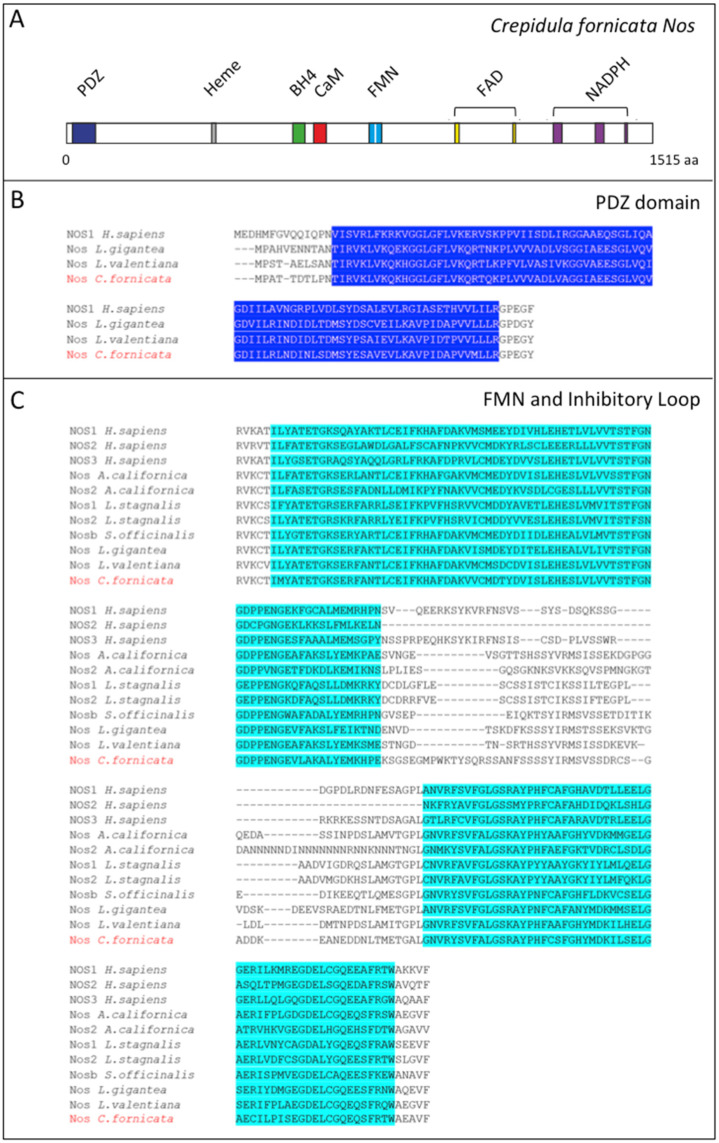
Protein domains characterization of Cfo-Nos (**A**) Schematic representation of Nos protein domains in slipper shell snail *C. fornicata*. Colour code: PDZ in blue, Heme in grey, BH_4_ in green, CaM in red, FMN in light blue, FAD in yellow, and NADPH in purple. The uncoloured sequence within the FMN domain is the inhibitory loop. (**B**) PDZ domain in *Cfo*-Nos as revealed in the alignment with *Homo sapiens* NOS1, *L. gigantea* and *L. valentiana* Nos. (**C**) FMN domain and the inhibitory loop in *Cfo*-Nos as revealed in the alignment with *Homo sapiens* NOS1, NOS2 and NOS3; *Aplysia californica* Nos and Nos2; *Lymnaea stagnalis* Nos1 and Nos2; *Sepia officinalis* Nosb; *L. gigantea* and *L. valentiana* Nos. The displayed alignment is focused on the FMN domain and the inhibitory loop.

Searches in *C. fornicata* embryonic and larval transcriptomes retrieved only one *Nos* ortholog gene in this species, encoding a 1515 amino acids long protein, although we cannot exclude the existence of a second gene expressed in adulthood, and not detected in this study. In order to characterize the inferred protein, we studied the domain composition of our candidate, by comparison with Nos from other species for which functional domains have already been described: human and other molluscs Nos [22]. We identified by comparative sequence analysis the binding domains for calmodulin (CaM), and the cofactors heme, tetrahydrobiopterin (BH4), flavin mononucleotide (FMN), flavin adenine dinucleotide (FAD) and nicotinamide adenine dinucleotide phosphate (NADPH) (Figure 1A). The protein alignment of *Cfo*-Nos with neuronal-type Nos from *Homo sapiens* (NOS1), *Lottia gigantea* and *Lehmannia valentiana* revealed the presence of the PDZ domain located from residue 11 to 94 (Figure 1B), considered a neuronal-specific feature [30]. Moreover, PDZ domain is responsible for the recruitment of neural Nos to NMDA receptors, which result in the ingression of calcium ions (Ca^2+^) to the cell that ultimately activates the protein via interactions with PDZ [31]. On the other hand, the sequence alignment of *Cfo*-Nos with human NOS1, NOS2, NOS3 and several mollusc Nos orthologs, highlighted the presence of the internal sequence within the FMN-binding domain (807-994 aa in human NOS1), called the inhibitory loop and located from 883 to 938 aa (Figure 1C). This element is responsible for the Ca^2+^ dependence, it inhibits intradomain electron transfer but, at specific Ca^2+^ concentration, CaM acts by displacing the autoinhibitory element allowing the enzymatic activity [32]. These two features together, the presence of the PDZ and the inhibitory loop, allow the classification of *Cfo*-Nos as a constitutive neuronal-type protein.

### 3.2. Cfo-Nos Expression during Development Suggests an Involvement in Shell Formation and Neural Specification

*C. fornicata* development starts with several cell cleavages following a very stereotypical program called spiral cleavage, leading up to gastrulation through epiboly. At mid-gastrulation (130 h post fertilization, hpf), the embryo starts elongation, and the blastopore, which will give rise to the future mouth, finishes closing while moving to the anterior region of the embryo. Once gastrulation is completed (140 hpf), organogenesis begins with the formation of a preveliger larva (150 hpf) that displays several characteristic structures like the ciliated velum (for propulsion and particulate food collection), the foot, the shell gland (that secretes the components required for forming the shell of the developing larva), the eyes, the neural primordia, and the gut. In a later stage, the preveliger larva suffers a visceral torsion (190 hpf) and the rest of the organs develop, giving rise to the veliger larva (200 hpf) with a defined nervous system, eyes, shell, digestive tract, and kidney, among other structures. Neuronal elements develop in early veliger stages prior to the appearance of any ganglia of the future adult central nervous system [33]. The detailed description of these neuronal elements in the veliger larva of *C. fornicata* has shown the presence of FMRFamide-like immunoreactive (LIR) cells as well as serotonin-LIR cells in the apical organ, a sensory structure considered to be involved in larval settlement, metamorphosis and locomotion [33]. Additionally, FMRFamide-LIR and serotonin-LIR projections in the velum and foot were also detected at the veliger larva [33]. As the veliger larva develops, peripheral FMRFamide-LIR and later catecholaminergic cells are located in the foot region, while catecholaminergic cells and processes were observed near the mouth [33].

**Figure 2:**
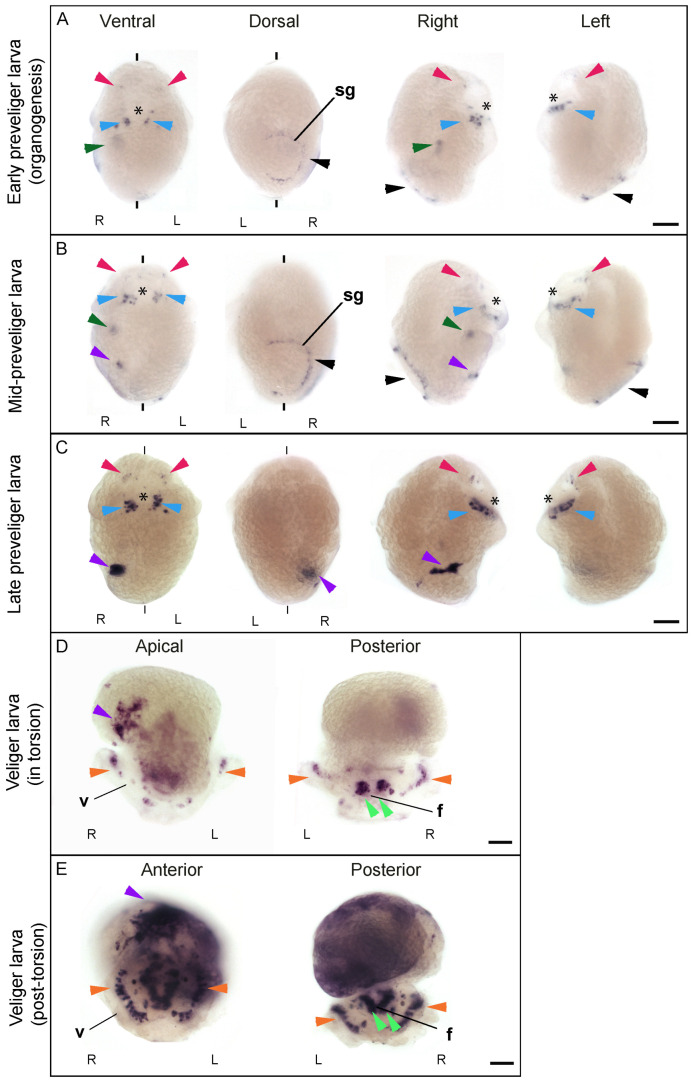
Spatiotemporal localization of Cfo-*Nos* expression in *C. fornicata* from preveliger to veliger larval stage. (**A**–**C**) show three sequential preveliger stages during development, respectively early (160 hpf), mid (170 hpf) and late (180 hpf). Preveliger larvae are shown in all four orientations, ventral, dorsal, right and left, to clarify interpretation of expression territories. (**D**,**E**) show two different veliger stages, torsion (190 hpf) and post-torsion (200 hpf) veliger larva stage, respectively. Panels D shows a larva in apical and posterior view, and panel E in anterior and posterior views. The asterisk shows the stomodeum. Arrowheads indicate: red, cephalic signals with apical and cerebral ganglions; light-blue, blastoporal nerve cord signals; black, shell gland signal; dark-green, right upper signal; purple, right lower signal; orange, sensorial organs at the velum; light-green, foot and catecolaminergic signals. f, foot; sg, shell gland; v, velum; R, right; L, left. The scale bar is indicated for each developmental stage and corresponds to 30 μm.

In *C. fornicata*, *Cfo*-*Nos* mRNA is detected during the development of neural elements, at the structure responsible for shell secretion (the shell gland) and the developing digestive tract and kidney (Figure 2). *Cfo*-*Nos* mRNA is first detected at the preveliger stage (160 hpf) (Figure 2A). At this stage, two discrete groups of cells start expressing *Cfo*-*Nos* symmetrically under the blastopore (future mouth), in the blastoporal nerve cord or circumblastoporal nerve cord (reviewed in [34]) (Figure 2A, blue arrowheads). In addition, a few more signals are detected in the area where the apical organ and the cerebral ganglions will form, on the anterior region of the head (Figure 2A, red arrowheads), which presumably correspond to the FMRF-amide and serotoninergic neurons that start to develop [33]. Additionally, *Cfo*-*Nos* expression is detected in a single band of cells surrounding the shell gland (Figure 2A, black arrowhead). Interestingly, the expression of *Cfo*-*Nos* does not completely surround the shell gland, but is asymmetric being absent in most of the left side. Finally, an internal signal can be distinguished exclusively on the right side of the embryo (Figure 2A, dark-green arrowhead), which corresponds to the domain of the developing kidney [33].

The expression in all these domains is maintained and gets even stronger as the organogenesis proceeds and the preveliger larva develops (Figure 2B). However, a new asymmetric signal is also detected exclusively on the right side at 170 hpf (Figure 2B, purple arrowhead), which corresponds to the hindgut [33].

In a later preveliger larva (180 hpf), close to the torsion event, most of these signals remain, like those on the anterior region of the head (Figure 2C, red arrowheads), on both sides of the blastopore (Figure 2C, blue arrowheads), and the hindgut domain (Figure 2C, purple arrowhead). In fact, these signals get stronger and increase the extension of their expression domain. However, the most anterior asymmetric signal on the right (Figure 2C, dark green arrowhead) and the incomplete ring around the shell gland are no longer detected (Figure 2C, black arrowhead). These results suggest that *Cfo*-*Nos* is involved in the first steps of specification or the segregation of the shell but is no longer required once it starts forming. Some other genes, like *Dpp* and *Nodal*, have been shown to regulate shell formation during the whole process [35,36,37].

At the transition to veliger larva, when torsion begins (190 hpf), the asymmetrical patch on the right side corresponds to the hindgut. This domain extends and moves dorsally due to the torsion process (Figure 2D,E, purple arrowhead). Interestingly, cells corresponding to the catecholaminergic neurons at the foot and mouth start expressing *Cfo*-*Nos* (Figure 2D,E, light-green arrowhead). It is also noteworthy that this bilateral signal maybe related with the formation of statocysts, since both are located in the same region in a bilateral manner. Additionally, *Cfo*-*Nos* expression is detected at the sensory buttons of the ciliated velum (Figure 2D,E, orange arrowhead), which correspond to the FMRF-amide, serotoninergic and catecholaminergic neurons [33]. In a veliger larva, when torsion is complete (200 hpf), *Cfo*-*Nos* expression is generalized in the visceral mass, especially strong on the intestine (Figure 2E, purple arrowhead).

The asymmetry of *Cfo-Nos* expression in the hindgut is probably due to the asymmetrical nature of this organ in *C. fornicata*. Based on our results we have no reason to think *Nos* has a role in left-right asymmetry during development, differently to other genes for which the expression pattern has been linked to the asymmetric distribution of body structures [26,35,38].

## 4. Conclusions

It has been previously demonstrated that Nitric Oxide acts as an endogenous inhibitor of metamorphosis in *C. fornicata*, through pharmacological inhibition experiments of Nos enzymatic activity [8]. Importantly, the expression of Nos was detected by immunohistochemistry with a universal anti-NOS antibody in the apical organ of young larvae, which is a neural structure conserved in many marine invertebrate larvae involved in their settlement, metamorphosis and locomotion [39]. Nevertheless, unspecific positive signals were detected in the digestive gland and stomach. In an attempt to complement those findings, we have carried out experiments aimed at the localization of *Nos* transcripts, instead of protein, in order to bypass possible cross reactions due to the use of a non-specific antibody. Furthermore, our goal has also been to look at the expression of *Nos* in *C. fornicata* through the entire embryo development, since there was evidence of *Nos* expression in pre-competent larvae (4–5 days post fertilization, dpf) and not in older competent larvae (10–20 dpf). While we do not detect expression of *Cfo-Nos* in early stages, we detect the transcripts at 165 hpf. This is in line with the fact that gradually declining NO concentrations in competent larvae is considered a prerequisite to enter metamorphosis. With this already known, we were rather interested to understand the contribution of Nos, and therefore NO, during organogenesis and, additionally, if it has a key role also in the organization of body structure, besides metamorphosis. 

Future research could be directed to perform loss-of-function and gain-of-function experiments aimed to understand the exact contribution of NO in *C. fornicata* development and metamorphosis. Although these in vivo approaches are not yet routinely executed in Crepidula, in this specific case it would be advantageous to suppress or overexpress the single copy *Cfo-Nos* gene as well as the availability of a developmental fate map in this species. In this perspective, it would be particularly interesting to also explore other possible NO functions besides the regulation of metamorphosis, especially its contribution to nervous system or shell gland secretion. Moreover, a related evo-devo aspect remains to be answered: what is the impact of NO in direct developer mollusc species, like for example in the sibling species *Crepidula* *atrasolea*? A future comparative study could answer this evo-devo question, and investigate if NO in species that do not metamorphose has a similar or different effect on neural specification and shell development.

## Figures and Tables

**Table 1:** Nitric Oxide signalling in molluscs. Survey of Nitric Oxide Synthases and NO signalling studied in molluscs, biological function and reference manuscripts.

*Ilyanassa obsoleta*	Metamorphosis (negative regulator)	[9]
*Crepidula fornicata*	Metamorphosis (negative regulator)	[8]
*Alderia willowi*	Metamorphosis (negative regulator)	[10]
*Mytilus coruscus*	Metamorphosis (negative regulator)	[7]
*Crassostera gigas*	Metamorphosis (negative regulator), Immune Response	[11]
*Chlamys farreri*	Immune Response	[14,15]
*Paphia malabarica*	Immune Response	[16]
*Sepia oficinalis*	Development, Neurotransmission	[12]
*Lymnaea stagnalis*	Neurotransmission, Chemosensory activation	[13]
*Aplysia californica*	Feeding, Chemosensory activation	[17]
*Lehmannia valentiana*	Olfaction	[18]
*Limax valentianus*	Olfaction	[19]
*Helix pomatia*	Olfaction	[20]
*Stramonita haemastoma*	Sensory Perception, Stress Response	[21]

## Data Availability

Not applicable.
